# Fiber-reinforced Composite Resin Bridge: A Treatment Option in Children

**DOI:** 10.5005/jp-journals-10005-1285

**Published:** 2015-04-28

**Authors:** Amit Gupta, Rama Krishna Yelluri, AK Munshi

**Affiliations:** Student, Department of Pedodontics and Preventive Dentistry, KD Dental College and Hospital, Mathura, Uttar Pradesh, India; Professor, Department of Pedodontics and Preventive Dentistry, KD Dental College and Hospital, Mathura, Uttar Pradesh, India; Professor and Head, Department of Pedodontics and Preventive Dentistry, KD Dental College and Hospital, Mathura, Uttar Pradesh, India

**Keywords:** Composite resin, Polyethylene fiber, Pontic, Resin bridge, Ribbond.

## Abstract

A number of treatment options, ranging from Maryland bridges to implants, are available for the replacement of congenitally or traumatically missing permanent anterior teeth. But, there are several limitations of these therapeutic options when they have to be used before the completion of the growth, particularly in children. Reinforcement of composite resins with polyethylene fibers significantly improves their mechanical properties. Fiber-reinforced composite (FRC) bridge can offer a good alternative to conventional treatment options in replacing a missing permanent anterior tooth until a more definitive prosthesis can be provided at the end of the growth period. The purpose of this article is to present a clinical case of a single tooth replacement utilizing noninvasive and metal free fixed FRC bridge in a 13 years old child as an interim treatment option.

**How to cite this article:** Gupta A, Yelluri RK, Munshi AK. Fiber-reinforced Composite Resin Bridge: A Treatment Option in Children. Int J Clin Pediatr Dent 2015;8(1):62-65.

## INTRODUCTION

The replacement of a single tooth, especially in a growing child, is always a challenge for the clinician. Reasons for tooth loss could be congenital absence, caries or trauma. Of these, traumatic injuries resulting in avulsion of the tooth is one of the common causes for tooth loss in the anterior region of the oral cavity. The various treatment options available in the management of this condition are: replantation of the avulsed tooth, removable partial denture, porcelain fused metal (PFM) bridge, resin-bonded fixed partial denture (Maryland bridge) and dental implants (immediate or delayed).

Long-term success of a replanted avulsed tooth is always questionable because of the resorption while more than half of the teeth are eventually lost because of ankylosis or Inflammatory resorption.^1^ Removable partial dentures (RPDs) are often the treatment of choice in a growing child until the permanent teeth have erupted, alveolar bone changes have decreased and the pulp chambers have receded to allow preparation for fixed replacement.^2^ But the lack of compliance in appliance wear and care by the young child is the greatest limitation of these appliances. Another contraindication for prosthetic replacement of this type is the presence of an anterior deep bite.^3^ Prosthesis like porcelain fused metal (PFM) is delayed until the gingival margins are reasonably stable and also rigid fixation of two teeth will lead to retardation of growth between the two teeth. Bonded Maryland bridges have also been used to replace missing tooth, but the unesthetic appearance provided by the metal framework, and the low bond strength between metal and the enamel makes it a less desirable option.^4-6^ The replacement of a missing permanent central incisor via a cantilever fixed partial denture could result in a compromised support and additional torsional forces on the abutment teeth.

On the contrary, dental implants are not recommended before the completion of growth period because unlike ankylosed teeth, they do not follow the growth of the facial bones and the risk of apical displacement is always there.^7,8^ Also, the high cost of implant prosthesis in replacing a missing tooth could limit its use, especially in the developing countries. Fiber-reinforced composites (FRCs) are resin-based materials containing fibers to improve their physical properties. These were introduced first in the 1960s by Smith when glass fibers were used to reinforce polymethyl methacrylates.^9^ Different fiber types, such as glass fibers, carbon fibers, kelvar fibers, vectran and polyethylene fibers have been added to composite materials to enhance their properties.^10^ Of these, polyethylene fibers in particular have been used for a number of clinical applications in dentistry *viz*: fixed space maintainer, endodontic post and core, splints, fixed partial dentures with a natural or artificial tooth as pontic, composite repairs and repair of a denture.^9,10^

This article describes a clinical case in which a FRC resin bridge utilizing an artificial tooth pontic was given for the purpose of long-term space maintenance as well as an interim treatment option in the maxillary anterior region until a fixed prosthesis can be planned at the end of the growth period.

**Fig. 1:**
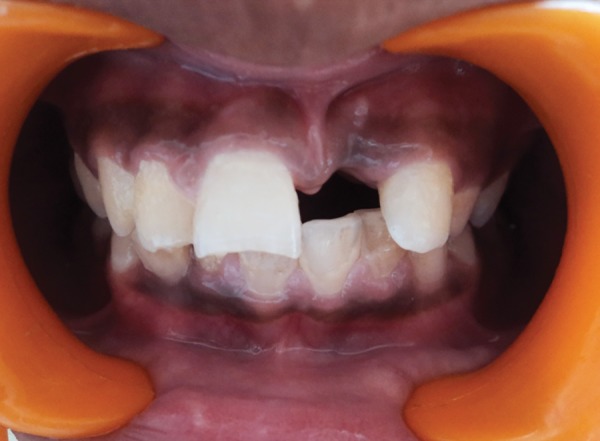
Preoperative frontal view of the missing maxillary left central incisor

**Fig. 2:**
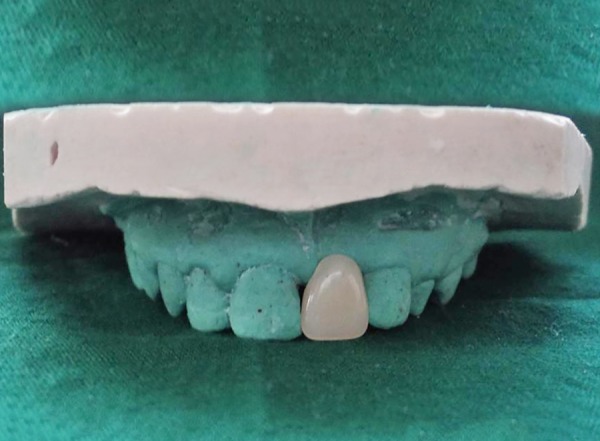
Selected acrylic pontic

**Fig. 3:**
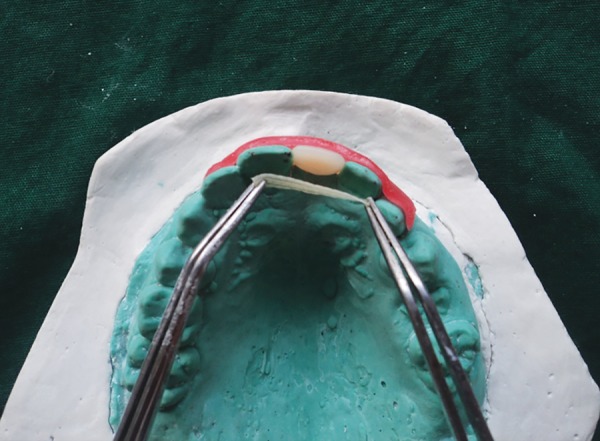
Polyethylene fiber being measured on the dental cast

## CASE REPORT

A 13 years old male child reported to the Department of Pedodontics and Preventive Dentistry, KD Dental College and Hospital, Mathura, with a chief complaint of missing tooth in the upper front teeth region since past 2 months. Medical history revealed no specific problem. His dental history indicated a traumatic fall, 2 months back, resulting in the avulsion of his maxillary left permanent central incisor. Intraoral examination revealed a completely healed socket in relation to maxillary left permanent central incisor and Ellis class I fracture of maxillary right permanent central and lateral incisor. Also, it was found that the dental midline of the patient did not coincide with the maxillary labial frenum (Fig. 1). An intraoral periapical radiograph of the concerned area presented with a healed bony socket in relation to the avulsed maxillary left central incisor. The patient’s oral hygiene status was fair. The age of the patient was not suitable for a fixed prosthesis and, after discussing all the treatment options with the patient and his parents, a FRC resin bridge was planned in the anterior region utilizing a natural or artificial tooth pontic. But, the limited options available in shade selection with extracted natural tooth led us to use an acrylic tooth pontic instead.

**Fig. 4:**
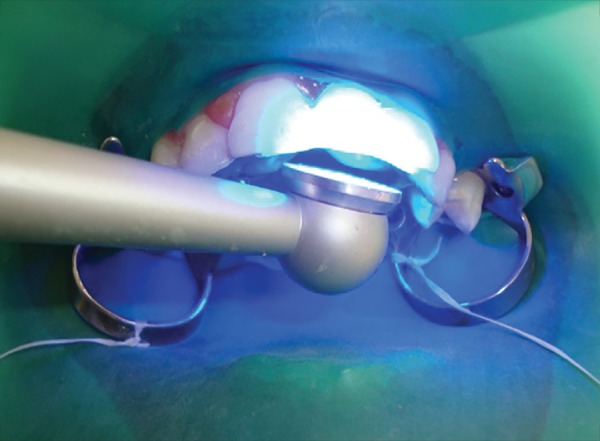
Photopolymerization of the FRC resin bridge

**Fig. 5:**
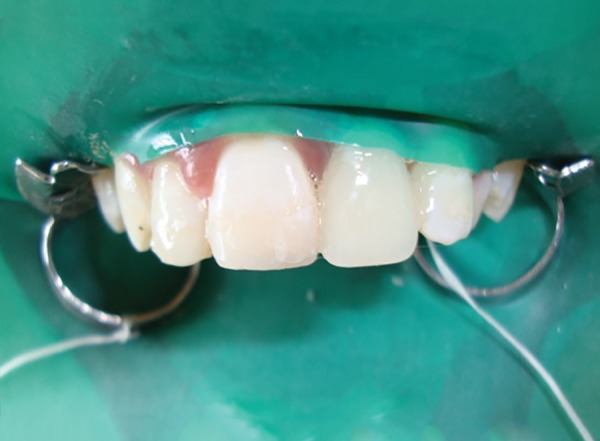
Labial view of the finished and polished FRC resin bridge

**Fig. 6:**
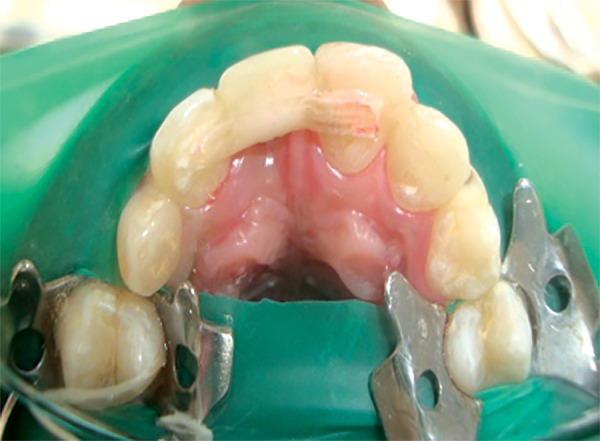
Palatal view of the FRC resin bridge

**Fig. 7:**
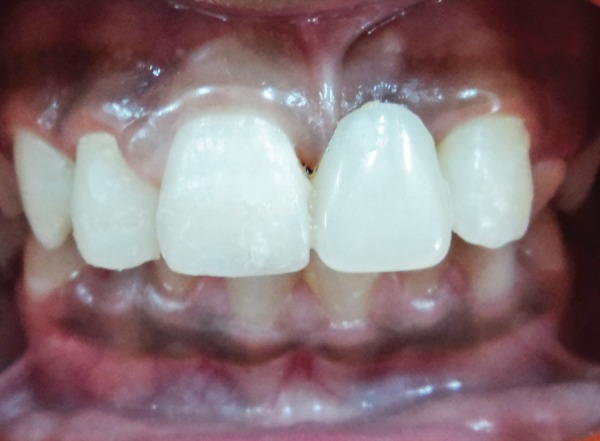
Final frontal view of the FRC resin bridge after the rubber dam removal

After oral prophylaxis, alginate impressions of the upper and lower arch were recorded, and the casts were poured in dental stone. Following this, shade selection was done under natural light and an acrylic tooth (Premadent, New Delhi, India) was selected (Fig. 2). Rubber dam was applied after administering local anesthesia and composite buildup of the maxillary right permanent central incisor and maxillary right permanent lateral incisor was carried out. Commercially available polyethylene fiber (Ribbond Inc., Seattle, WA, USA) was utilized in this case and the length of the fiber was measured on the dental cast (Fig. 3). The fiber length was kept just short of the distoproximal surface of the abutment teeth. Polyethylene fiber of 20 mm in length and 2 mm in breadth was wetted with an unfilled adhesive resin (Adper^™^, Single Bond, 3M-ESPE, St Paul, MN, USA) and kept in a dappen dish away from dental light until use. The lingual enamel surfaces of the abutment teeth were etched with 37% phosphoric acid (Scotchbond^™^, 3M, ESPE, USA) for 15 seconds, rinsed with water, air dried, and a single coat of adhesive resin was applied and photopolymerized for 40 seconds. Polyethylene fiber was then adapted to the middle third of the lingual surfaces of the abutment teeth and photopolymerized for 40 seconds. A small amount of restorative composite resin (Tetric N-Collection, Ivoclar Vivadent, Liechtenstein) was applied to the facial surface of the polyethylene fiber that would be contacting the acrylic pontic. Subsequently, the pontic was positioned on the polyethylene fiber in proper alignment and photopolymerized for 40 seconds. The restorative composite resin was also applied on the palatal aspect of the fiber in contact with pontic and photo-polymerized for 40 seconds (Fig. 4). The occlusion was checked, and final finishing and polishing was done. The final result was a well-adapted bridge with good esthetic result (Figs 5 to 7).

## DISCUSSION

Composites that are reinforced with polyethylene fibers can result in materials with enhanced mechanical properties,^11,12^ i.e. stiffiness, strength, toughness and less fatigue.^13^ Fibers produce a load-enhancing effect on the brittle composite materials by acting as the stress-bearing component and by crack-stopping or crack-defecting mechanisms.^12,14^ Fixed FRC bridges offer a suitable alter-native to replace a missing permanent anterior tooth, especially in a growing child until a fixed prosthesis can be provided at the end of growth period. Advantages of this design include bondability, chairside ease of fabrication and repairability.^6^ It is both economical and less time consuming as the fabrication can be performed in a single appointment. The design is noninvasive and reversible so other conventional treatment options always remain open. Studies by Unlu and Belli^15^ and Freilich^16^ have reported a mean survival period of 3 and 4.5 years respectively, for fixed FRC bridges which make it a suitable interim treatment option for replacing missing permanent anterior teeth in child patients until a definitive restoration can be provided. However, disadvantages include difficulty in maintaining the oral hygiene and its questionable ability to withstand heavy masticatory load. Hence, fixed FRC bridges might offer a metal-free and clinically acceptable option for interim replacement of a missing permanent anterior tooth, but further studies are needed to verify the success of these FRC bridges.

## CONCLUSION

The case presented in this article suggests an interim treatment option for the replacement of missing anterior tooth in young children. This technique does not require any tooth reduction and could be repaired, modified or removed from the abutment teeth without any damage to the sound tooth structure.
